# Evaluate the forensic efficiency parameters of the *D13S317* gene in closely related family members in Gondar town, Northwest Ethiopia

**DOI:** 10.1016/j.jgeb.2025.100628

**Published:** 2025-11-30

**Authors:** Betelhem Abebe Begashew, Temesgen Mitiku Yeshanew, Wagaw sendeku, Nega Birhane

**Affiliations:** a Department of Biotechnology, Dambi Dollo University, P.O. Box 260, Dambi dollo, Ethiopia; b Department of Medical Biotechnology, Institute of Biotechnology, University of Gondar, P.O. Box 196, Gondar, Ethiopia

**Keywords:** DNA profiling, *D13S317* locus, STR, Allele frequency, Discrimination power

## Abstract

**Background:**

Forensic DNA typing has become widely accepted in criminal investigations, familial relationship testing, and identification. This process currently relies on the analysis of short tandem repeat (STR) markers. This is because DNA profiling serves as a highly effective means of identifying individuals on the basis of their distinct genetic characteristics. DNA evidence can also be utilized to clear innocent suspects of any wrongdoing. Recent technological advancements in DNA profiling, such as the implementation of statistical methods, have increased the precision of this tool in forensic investigations.

**Objective:**

The main objective of this study was to evaluate the forensic efficiency parameters of the *D13S317* marker in closely related family members in Gondar town, Northwest Ethiopia.

**Methods:**

To conduct this study, 87 blood samples were collected in EDTA tubes from various kebeles in Gondar town between October and December 2024. DNA was extracted via the salting-out method, and PCR amplification was performed with forward and reverse primers. After amplification, the PCR products were analyzed on a 1.8 % agarose gel and visualized with a gel documentation system.

**Results:**

The discrimination power, random match probability, typical paternity index, polymorphism information content, and genetic diversity of the D13S317 marker were 0.77, 0.232, 0.5, 0.73 and 0.77, respectively. Five alleles were also confirmed, with the 192 bp allele exhibiting the highest frequency at 33 %, whereas the 176 bp allele had the lowest frequency at 5.7 %.

**Conclusion:**

The D13S317 marker has average discrimination power, making it a valuable tool for DNA profiling, paternity testing, and population genetics studies.

## 1 Introduction

In modern forensic science, DNA analysis has become the cornerstone tool in criminal investigations, establishing sibling relationships, responding to mass disasters, and conducting maternity and paternity testing.^1^ Emerging modern DNA analysis technologies such as gel or capillary electrophoresis, next-generation sequencing (NGS), rapid DNA analysis, microfluidics/lab-on-a-chip technologies, AI-driven analysis, forensic genealogy, and 3D genomics accelerate and increase the accuracy of DNA analysis.^2^ These advancements enable forensic scientists to extract meaningful information from complex DNA mixtures.^1^ However, this technology is not yet universally accessible in economical poor country, because of the high costs associated with the equipment and the specialized training required for effective utilization.Table 1.Table 2.Table 3..

**Table 1:** Thermal cycle conditions used to perform PCR.

Initial denaturation Step	Denaturation Step	Cycle	Annealing	Extension	Final extension	PCR product (bp)
95 °C for 4 min	94 °C for 45 s	35	53 °C for 1 min	72 °C for 1 min	72 °C for 10 min	176–––192

**Table 2:** Sociodemographic characteristics of the study participants.

No/	Variable	Study participant	Number (%)
1.	Gender	Male	56 (64.4)
		Female	31(35.6)
2.	Age	18–25	16 (18.4)
		26–35	23 (26.4)
		36–45	31(35.6)
		>46	17 (19.5)
3.	Marital status	Single	24 (27.6)
		Marriage	58 (66.7)
		Divorced	5 (5.7)
4.	Job	Formal Employment	21(24.14)
		Informal Employment	37(42.5)
		Self-Employed	29(33.3)
5.	Educational status	No Formal Schooling	9(10.3)
		Primary and secondary Education	46(52.9)
		Tertiary Education	32(36.8)

**Table 3:** Alleles, number of observations, frequency and percentage (N = 87).

**No/**	Allele	Observation	Frequency
	176	5	5.747 %
	180	12	13.793 %
	184	19	21.839 %
	188	22	25.287 %
	192	29	33.333 %
*p*	0.001		

In recent years, numerous molecular markers have been employed to investigate genetic diversity, criminal investigations, and maternity and paternity testing.^3^ Common molecular markers utilized in forensic applications include short tandem repeats (STRs), restriction fragment length polymorphisms (RFLPs), STR-single nucleotide polymorphisms (STR-SNPs), Y-short tandem repeats (Y-STRs), and the Y and X chromosomes.^4^, ^5^ Short tandem repeats (STRs), classified as second-generation molecular markers, consist of tandem repeats of 1 to 6 nucleotides accompanied by conserved flanking sequences.^6^ Microsatellite markers are extensively utilized in the analysis of population genetic diversity, examination of genetic structure, identification of parentage, development of genetic linkage maps, identification of crime scenes and identification of victims.^7^, ^8^ .

Currently, PCR-based autosomal STRs represent the standard methodology employed in forensic laboratories for identity verification and individualization.^9^, ^10^ The selected autosomal STRs for DNA profiling consist of tetra- and pentanucleotide core repeat STRs that are unlinked and situated on distinct sets of chromosomes.^11^ STRs account for approximately 3 % of the human genome.^12^, ^13^ STR profiling is an invaluable tool in forensic science, as it provides a high level of discriminability and requires only trace amounts of DNA for the creation of complete or partial profiles.^14^, ^15^ The highly polymorphic nature, reproducibility, sensitivity, specificity, reliability and high signal‒to‒noise ratio of the STR make it ideal for most forensic uses to identify individuals, identity resolution, criminal investigations and the identification of missing persons.^16^ STR has also been regularly used in murder investigations,^17^ sexual assault cases^18^ and cell line identification.^19^ This is strongly supported by numerous population genetic studies that have investigated allele frequencies and forensic parameters such as the paternity index (PI), heterozygosity, power of discrimination, power of exclusion, polymorphic information content, and matching probability of autosomal STRs.^20^, ^21^, ^22^ .

In 2017, the FBI increased the number of core short tandem repeats (STRs) from 13 to 20. The expanded list includes the following STRs: CSF1PO, FGA, THO1, TPOX, VWA, *D3S1358*, *D5S818*, *D7S820*, *D8S1179*, *D13S317*, *D16S539*, *D18S51*, *D21S11*, *D1S1656*, *D2S441*, *D2S1338*, *D10S1248*, *D12S391*, *D19S433*, *D22S1045*, and amelogenin.^9^, ^23^ Thus, CODIS significantly improves the search for suspects and the identification of case connections on a national scale.^24^ Since this change, numerous countries have adopted similar practices by establishing their own combined DNA index system (CODIS) and databases.^23^ Consequently, validated combined DNA index system (CODIS) short tandem repeats (STRs), which necessitate a minimal quantity of DNA, along with polymerase chain reaction (PCR) and its statistical strengths, are routinely employed to identify human remains, determine paternity, or match biospecimens from suspected crime scenes.^11^ .

Despite the potential applications of CODIS loci for forensic purposes, their use in Ethiopia remains rare. This gap arises primarily from systemic challenges, including financial constraints, a lack of necessary laboratory equipment, and the lack of a well-organized forensic laboratory staffed with a sufficient number of trained professionals. This study aims to evaluate the forensic efficiency parameters of the D13S317 locus, focusing on power of discrimination (PD), genetic diversity (GD), allele frequency, and other parameters within the population of Gondar town.

## 2 Material and method

### 2.1 Study area

**Fig. 1:**
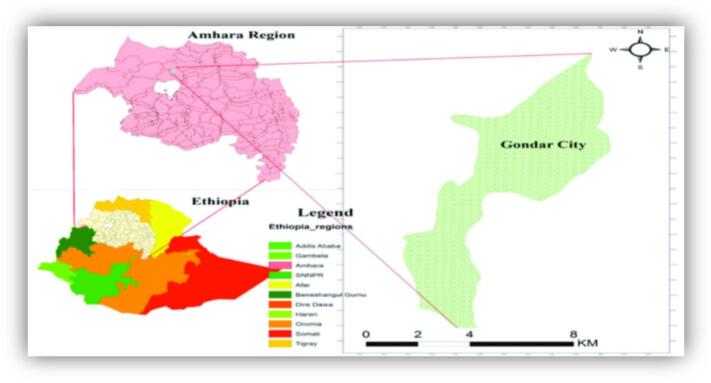
Map of the study area (Gondar city).

This study was conducted in Gondar town, Amhara region, Northwest Ethiopia. Gondar city is approximately 750 km from Addis Ababa, the capital city of Ethiopia, at 12°30  N–12°39′N–37°24′E–37°29′E (Fig. 1). The total population of Gondar town is estimated to be 327,900. This city is among the ancient and most populated cities in the country. Currently, Gondar city has one referral hospital and eight government health centers.

### 2.2 Study design

This research utilized a laboratory-based cross-sectional design to evaluate the *D13S317* marker for forensic purposes in Gondar town.

### 2.3 Study period

The study was conducted in the Amhara region, Northwest Ethiopia, from October 2024 to September 2025.

### 2.4 Source of the population

The source population for this study comprised residents of Gondar town. The participants were selectively enrolled on the basis of the availability of documented family pedigree data. Prior to inclusion, informed consent was obtained from all the subjects for the collection of blood samples, DNA profiling, and their use in subsequent research.

### 2.5 Study population

The study population comprised closely related individuals from Gondar Town, Northwest Ethiopia, including parent‒child pairs, siblings, and extended relatives. A target sample size of 87 participants from multiple families was determined to ensure sufficient genetic diversity and representation of the local population.

### 2.6 Inclusion criteria

Eligible participants were individuals who provided informed consent for DNA sampling and met one of the following criteria: (a) having a confirmed biological relationship with another participant (e.g., parent‒child, sibling pairs) or (b) being a healthy volunteer from the community.

### 2.7 Exclusion criteria

The exclusion criteria were as follows: (1) individuals with no confirmed or uncertain familial relationship to the core pedigree; (2) potential participants unable to provide informed consent; and (3) minors under the age of 18, consistent with ethical guidelines for genetic research and the standard practice of prioritizing adult profiles in forensic databases.

### 2.8 Study variables

The primary independent variables were the alleles identified at the *D13S317* locus and the nature of the familial relationship (e.g., parent‒child, siblings). Demographic characteristics, including age and sex, were also considered independent variables. The dependent variables included key forensic genetic parameters: heterozygosity (H), power of discrimination (PD), probability of matching (PM), polymorphic information content (PIC), and the paternity index (PI).

### 2.9 Sample size and sampling technique

The study participant sample size was determined via the following statistical formula:

N $=\frac{(Z^{2}.P\left(1-P\right)}{E^{2}}$ where N = the minimum required number of participants (N),

Z score (Z) = standard normal distribution value at the 95 % confidence level, which is 1.96; estimated proportion (p) = 0.1; and margin of error (E) = 0.05 (the maximum acceptable error).^25^ However, due to a lack of voluntary participants and significant financial constraints, recruitment and data collection were limited. Within these constraints, a nonrandom purposive sampling technique was used to select all eligible participants who were accessible and agreed to participate during the study period, resulting in a final sample size of 87.

### 2.10 Sample collection and preparation

Blood samples were collected from voluntary families, which included fathers, mothers, children, and relatives such as uncles, aunts, cousins, or grandparents. Each sample was labeled accordingly. In total, 87 blood samples were collected from various families in Gondar town, Northwest Ethiopia. Additionally, 16 blood samples for the positive control were collected from Yismala Kebele in North Gojjam and transported to the University of Gondar Molecular Laboratory in an icebox. The entire study was conducted in the molecular biology laboratory at the Institute of Biotechnology, University of Gondar. The collected blood samples were stored at −20 °C until use.

### 2.11 DNA Extraction

DNA was extracted from blood samples via a salting-out method based on the procedure described by.^26^ In brief, 900 µl of TKM 1 and 50 µl of 1x Triton-X were added to 300 µl of blood in an autoclaved 1.5 ml Eppendorf tube. The mixture was incubated at 37 °C for 5 min in a water bath and then centrifuged at 8000 rpm for 3 min, after which the supernatant was discarded. This process was repeated 2–3 times, with decreasing amounts of 1x Triton-X until red blood cell lysis was complete and a white pellet of white blood cells was obtained. Next, 300 µl of TKM 2 and 40 µl of 10 % SDS were added to the cell pellet, mixed thoroughly, and incubated at 37 °C for 5 min. After incubation, 100 µl of 6 M NaCl was added, and the mixture was vortexed to precipitate the proteins. The cells were then centrifuged at 8000 rpm for 5 min, and the supernatant was transferred into a new Eppendorf tube containing 300 µl of isopropanol. The DNA was precipitated by slowly inverting the Eppendorf tube. The tubes were subsequently centrifuged at 8000 rpm for 10 min to pellet the DNA. After the supernatant was discarded, 400 µl of 70 % ethanol was added, and the mixture was mixed gently to remove any excess salt. The tubes were centrifuged again at 8000 rpm for 5 min to pellet the DNA.

The supernatant was discarded, and the DNA was air-dried. Once thoroughly dried, 50 µl of TE buffer was added to dissolve the DNA. The quality and concentration of each extracted genomic DNA sample were assessed via gel electrophoresis and a Nanodrop 2000C and then stored at −20 °C until further use.

### 2.12 PCR

DNA amplification was performed via conventional PCR with forward and reverse primers on the basis of a previous protocol.^27^ Each reaction consisted of 2 µl of template DNA in a total volume of 20 µl, which included 10 µl of master mix, 7 µl of ddH2O, and 0.5 µl of each designed forward 5′ ACAGAAGTCTGGGATGTGGA 3′ and reverse 5′GCCCAAAAAGACAGACAGAA 3′.^28^ Nuclease-free water was included as a negative control, whereas samples collected from North Gojjam served as a positive control.

### 2.13 Gel electrophoresis

The PCR products were analyzed on a 1.8 % Agarose gel stained with Ethidium bromide via 1x TBE buffer and loading dye. A 50 bp DNA ladder was loaded alongside 10 µL of the PCR products to estimate the band sizes. Electrophoresis was conducted at 100 V for 45 min, and the products were visualized via a gel documentation system.

### 2.14 Data analysis

After all the relevant data were collected, the forensic efficacy parameters, including the power of discrimination (PD), random match probability (PM), genetic diversity (GD), polymorphism information content (PIC), and typical paternity index (TPI), were analyzed via STR analysis for forensics (STRAF), which was developed by Gouy and Zieger.^29^ The results were considered statistically significant at P < 0.05.

### 2.15 Data quality control

The study evaluating the forensic efficiency of the D13S317 marker among closely related family members in Gondar, Ethiopia, was supported by a comprehensive quality control plan that ensured accuracy and reliability. This plan included pre-analytical checks, such as proper sample collection, labeling, transport, and storage; analytical controls, including DNA purity verification, PCR optimization, and gel electrophoresis validation; and post-analytical validation, which involved double data entry and forensic parameter calculations via the STRAF.

## 3 Results

### 3.1 Sociodemographic characteristics

In this study, 87 blood samples were collected in Gondar town from different Kebeles. Among these samples, 64.4 % were from male participants and 35.6 % were from female participants (table 2). Most participants were married (66.7 %) and aged between 36 and 45 years. Employment status indicated a dominance of informal work at 42.5 %, followed by self-employment at 33.3 % and formal employment at 24.1 %. Educational status varied, with 52.9 % having primary or secondary education, 36.8 % holding tertiary qualifications, and 10.3 % not having formal schooling.

### 3.2 Forensic efficiency parameters

In genetic studies, evaluating the significance of DNA typing results through statistical analysis is essential. Implementing a statistical approach in forensic sciences enhances the understanding of the value of the obtained results. The parameters calculated to assess the weight of the data for this paper include the power of discrimination (PD), typical paternity index (TPI), and polymorphism information content (PIC), among others. Table 4 presents the forensic efficiency parameter values for the D13S317 locus.

### 3.3 Allelic frequency

**Fig. 2:**
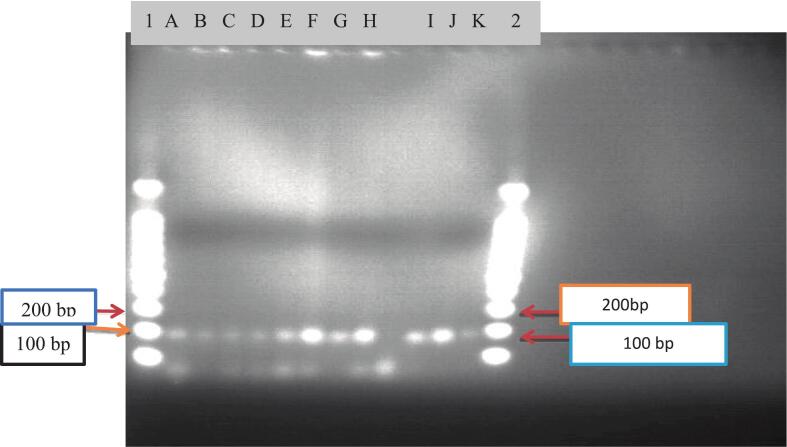
PCR-amplified agarose gel image of the D13S317 locus. Lane L1 is a 50 bp DNA ladder, Lane A, B, C, E, F, and H 184 bp. Lane D, G, I, and P 176 bp, and others lane are 180 bp.

**Fig. 3:**
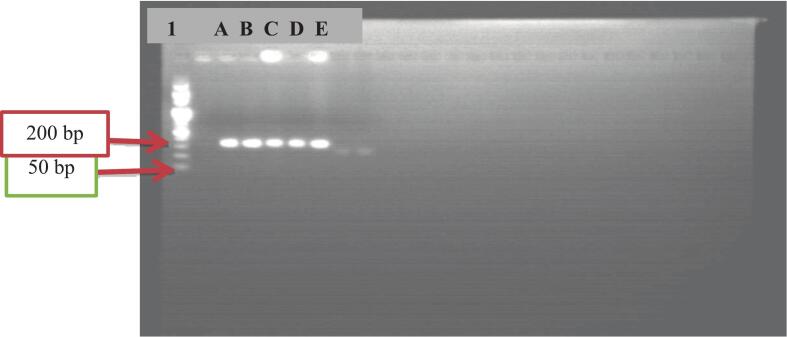
PCR-amplified agarose gel image of the D13S317 locus.

In this study, five different alleles were identified. The most prevalent allele was the 192 bp allele, with a frequency of 33.3 %as shown figure 3, whereas the 176 bp allele had the lowest frequency at 5.7 % as shown figure 2. The remaining alleles were 180 bp, 184 bp, and 188 bp long, with frequencies of 14 %, 22 %, and 25 %, respectively. Marriages between individuals of different ethnicities increase genetic variation and allele heterozygosity. This is supported by studies such as,^30^ which indicate that most of the modern population is a secondary race formed from a mixture of primary races, resulting in significant variations within each ethnicity.

Lane L2 is a 50 bp DNA ladder, Lane A-C and E-H 192 bp, Lane D and I-K 188 bp.

### 3.4 Discrimination power

The discrimination power of the *D13S317* locus in this study was 77 % (Table 4), which is lower than those from previous studies in Mozambique (87 %),^31^ Angola (86 %),^32^ Equatorial Guinea (86 %),^33^ India (88 %),^34^ Rwanda (88 %),^35^ Egypt (91 %),^36^ Panamanian (92.8 %)^37^ and Argentina (93 %).^38^ .

**Table 4:** Forensic efficiency parameters for the *D13S317* locus in the Gondar population.

Parameters	PD	GD	PIC	PM	PI
**D13S317** value	0.77	0.77	0.73	0.232	0.5

Abbreviations: PD, power of discrimination; GD, genetic diversity; PIC, polymorphic information content; MP, match probability; PI, paternity index.

Furthermore, the discriminating power of the D13S317 marker was lower than that of other STR loci, such as *D8S1179* (0.952), *D2S1338* (97 %), and FGA (97 %).^39^ These differences can be attributed to various factors, including genotyping methods, sample size and population structure. The discrimination power values for autosomal loci range from 75 % to 96 %, making them suitable for various forensic applications.^40^, ^41^ On the basis of the results of this study, the D13S317 locus can be reliably used to establish DNA-based tests for the Gondar town population for any forensic purpose.

### 3.5 Genetic diversity

With the advent of genomic tools, the use of molecular markers has become the preferred method for assessing genetic diversity. This approach involves studying variation among genotypes at the DNA/RNA level. The results obtained for the genetic diversity can be seen in Table 4. The genetic diversity of the population based on D13S317 loci was 77 %; this result is lower than that of studies conducted in Egypt (79 %),^36^ central India (82 %),^42^ and India (84 %).^39^ However, the results of this study was higher than those of studies conducted in Mozambique (72 %),^31^ Angola (71 %),^32^ Equatorial Guinea (70 %)^33^ and India (73 %).^34^ .

### 3.6 Match probability

The significance of a single-source DNA profile is evaluated primarily by calculating the random match probability (MP), which estimates the likelihood that a random individual from the population shares the same DNA profile. For the D13S317 loci, the match probability was determined to be 23 % (Table 4). This figure is notably higher than those reported in previous studies: 12 % in India,^34^ 7 % in Panama^37^ and 7.4 % in the Sikh population.^42^ .

### 3.7 Typical paternity index

The typical paternity index measures how much more likely a potential father is to be the actual father compared with a randomly selected man from the population, on average.^43^ In this study, the typical paternity index value for the D13S317 locus was 50 %. A higher typical paternity index for an STR marker signifies its greater utility in paternity testing within a population.

### 3.8 Polymorphism information content (PIC)

In this study, the polymorphism information content (PIC) for D13S317 was 73 %. This value is higher than the previously reported PIC for Rwandans (69 %)^35^ and West Bengal, India (72 %).^39^ However, it is lower than the PIC observed in other populations: 78 % in the Sikh population,^42^ 79 % in Panamanian populations,^37^ 77 % in West Bengal, India,^39^ 92 % in Beninese populations,^44^ and 81 % in the Rajbanshi population in India.^39^ .

## 4 Discussion

This study demonstrates the forensic application of the *D13S317* marker in the Gondar population by evaluating key forensic efficiency parameters and allele frequencies specific to this demographic. These findings highlight the significance of this marker for accurate individual identification and criminal investigations. As a result, the *D13S317* marker has emerged as an invaluable tool for DNA-based forensic analyses, enhancing the accuracy and reliability of genetic profiling.

Allele homozygotes and heterozygotes can be inherited from each parent, or the same pair of alleles can be inherited from both parents.^45^ According to that condition, a DNA test from allele patterns has a probability of paternity of 99.9 % or greater; if the patterns do not match two or more markers, then it can be excluded as biological evidence.^46^ The results of this study revealed the presence of homozygous alleles in the population at the *D13S317* locus. A study conducted in Pakistan^28^ also revealed that all alleles were homozygous. In contrast, a study in Indonesia^47^ identified heterozygous alleles at the *D13S317* locus. This difference primarily arises from population history (such as migration), cultural practices (including inbreeding), technical factors (such as genotyping methods), and sample collection.

**Fig. 4:**
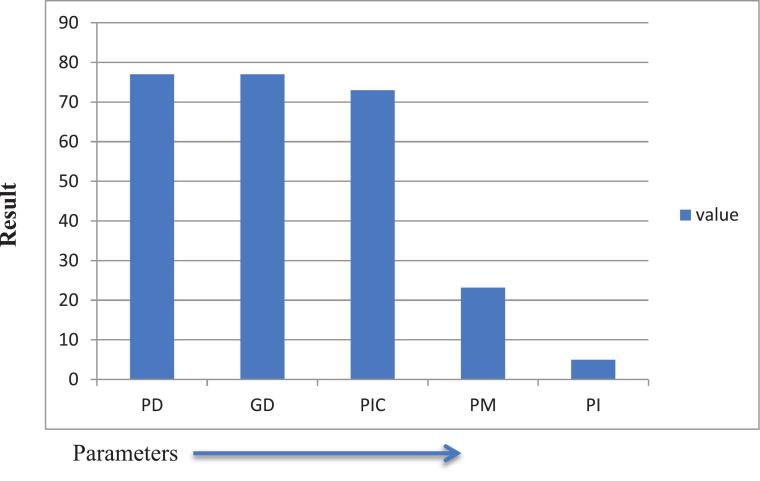
Overall forensic efficiency parameters of the *D13S317* marker GD: genetic diversity, PIC: polymorphism information content, PM: match probability, PD: power of discrimination, PI: paternity index.

The genetic history of a population is evident in the genomes of its members. A commonly utilized and efficient description of genetic data is the allele frequency spectrum, which reflects the distribution of allele frequencies across populations.^48^, ^49^ In this study, five alleles were identified at the *D13S317* locus, corresponding to repeat numbers 176, 180, 184, 188, and 192 (Fig. 2, Fig. 3). Among these alleles, allele 192 had the highest frequency at 33 %, whereas allele 176 had a lower frequency of 5.7 %. Notably, allele 192 was also reported as the most dominant allele in Kenya,^50^ Bosnian,^51^ Saudi Arabia^52^ and Panamanian.^37^ The primary reasons for the variation in allele frequency are evolutionary history, genetic drift, and natural selection.^53^ Additionally, allele frequency is influenced by the type of DNA genotyping methods^54^ as well as the sample size of the study recruited. A key limitation of this study is its reliance on gel electrophoresis, which restricts the identification of rare alleles, detection of potential stutter bands, and precise quantification of allele frequencies in small sample sizes.Fig. 4..

Short tandem repeat markers are crucial to human genetics because of their significant variability, which makes them vital for identifying individuals in criminal investigations and for distinguishing between populations and human migration patterns.^55^ In this study, the discrimination power of the *D13S317* locus was 77 %. This value is lower than that reported in previous studies from Sri Lankan (93.8 %),^56^ Rwanda (87 %),^57^ Argentina (93 %),^58^ Russia (93 %),^59^ and Austria (91 %).^36^ In contrast, the discriminatory power of this locus was greater than that reported in Iraq (55 %)^60^ and in Mozambique (71 %).^61^ Additionally, it is less comparable to results from Kenya (89 %).^50^ Several key factors contribute to the variation in discrimination power (DP) of the *D13S317* locus across different countries. The primary reasons include differences in local allele frequencies, the size and composition of the studied populations, the effects of genetic drift on laboratory techniques, and the specific characteristics of the genetic locus.

The present study assessed genetic diversity among Gondar populations on the basis of the *D13S317* locus. A value of 77 % confirms that D13S317 is highly informative for genetic studies, forensics, and ancestry analyses owing to its significant variability within the population. The high diversity at *D13S317* may be attributed to the combination of high mutation rates, gene flow, and genetic drift.^62^ Comparable results were observed in Egypt, with a value of 78.8 %,^63^ Kenya, with a value of 71 %^50^; and Saudi Arabia, with a value of 76 %.^52^ However, the results of this study were lower than those of a study conducted in Nigeria (92 %).^64^ In contrast, our result was higher than that reported from Zimbabwe (71 %).^65^ .

The PIC result for D13S317 in this study was 73 %, indicating a high level of polymorphism at this locus and its significant contribution to genetic variation in the Gondar population. This result is comparable to the values of 75 % reported in Egypt^63^, 72 % reported in Morocco^66^ and 72 % reported in Saudi Arabia.^52^ However, this value is higher than the 69 % reported in Kenya,^50^ 66 % reported in Rwanda,^57^ and 67 % reported in South Africa.^67^ In contrast, the PIC value of the D13S317 marker in this study was lower than that reported in Nigeria, at 79 %.^64^ The differences in *D13S317* PIC values across studies can be attributed primarily to variations in population genetic structure,^68^ sample size, and methodology.

The probability of matching was also measured at 23 %; this result was in line with that in India (24 %).^69^ The results of this study was higher than those of studies conducted in Nigeria (6 %),^64^ Kenya (11 %),^50^ Botswana (12 %),^70^ Rwanda (13 %),^57^ Zimbabwe (14 %),^65^ and Saudi Arabia (10 %).^52^ However, this percentage was lower than that reported in a study conducted in Sri Lanka (62 %).^56^ The study sample selected, along with population genetics and allele frequencies, is the primary factor influencing this figure. 0.119.

In this study, the typical paternity index (TPI) for the STR marker D13S317 was 50 %, demonstrating the strong utility of the marker in confirming biological relationships in paternity investigations within the Gondar population. However, the results of this study were lower than those of studies conducted in Botswana (68 %),^70^ Sri Lanka (79 %)^56^ and Nigeria (78 %).^64^ .

At present, there is no public or centralized STR database available for the Ethiopian population. Although no research has yielded significant insights into the genetic diversity of the Gondar population in Northwest Ethiopia, which includes data on allele frequencies for the STR *D13S317* marker, this information remains scattered and has not been organized into a cohesive, accessible database. Our study aims to fill this crucial void by gathering and analyzing an STR *D13S317* marker from the Gondar population. This initiative will not only improve the accuracy of forensic genetic profiling but also greatly enhance the interpretation of genetic data in forensic investigations, especially within the context of the Gondar town population in Northwest Ethiopia.

Moreover, the incorporation of genetic, historical, demographic, and anthropological data would create a more thorough understanding of the genetic landscape, thereby boosting forensic capabilities and providing valuable insights into Gondar town in Northwest Ethiopia and beyond.

## 5 Conclusion

This study assessed the forensic efficiency parameters of the *D13S317* locus in closely related individuals from Gondar town, Northwest Ethiopia. The results demonstrated a discrimination power of 77 %, a polymorphism information content of 73 %, and a random match probability of 0.232, indicating moderate discriminatory capability for forensic and paternity applications. Five alleles were identified, with the 192 bp allele being the most frequent (33 %) and the 176 bp allele the least frequent (5.7 %). While the locus exhibits sufficient discrimination power for preliminary forensic use, its intermediate efficiency parameters suggest that it should be combined with additional short tandem repeat (STR) markers and a sophisticated DNA typing method to increase reliability in kinship analysis and criminal investigations within this population. Further studies with larger sample sizes, modern genotyping methods such as Sanger or next-generation sequencing and expanded STR panels are recommended to improve the accuracy of forensic identification in the region.

## Data availability

The data used to support the findings of this study are included within the articled and attached as supplementary material.

## Ethical clearance

This study received ethical approval from the University of Gondar Institute of Biotechnology and ethical committee (IREC) under ethical clearance No. IoB/607/01/2025. Informed consent was obtained from all participants prior to their involvement. They were informed about the study's objectives and procedures, and their agreement was documented through signed consent forms.

## Authors’ Contributions

BA participated in the process of searching and selecting articles. TM conducted the statistical analysis and interpreted the data. All authors collaborated to prepare the draft manuscript also made revisions. TM, WS and NB completed the final version of the manuscript and submitted it to the journal. All authors read and approved the final manuscript before submission.

## CRediT authorship contribution statement

**Betelhem Abebe Begashew:** . **Temesgen Mitiku Yeshanew:** Writing – review & editing, Methodology, Formal analysis. **Wagaw sendeku:** Writing – review & editing. **Nega Birhane:** Supervision, Validation.

## Informed consent

Written informed consent was obtained from all study participants before the study started. The privacy rights of study subjects were protected. It was carried out in line with the ethical standards laid down in the Declaration.

## Funding

The authors received no financial support for the research and/or authorship of this article that it is not possible to ensure complete anonymity, and someone may be able to recognize me. However, by signing this consent form I do not in any way give up, waive or remove my rights to privacy. I may revoke my consent at any time before publication, but once the information has been committed to publication (“gone to press”), revocation of the consent is no longer possible.
